# Association of the SNPs in CCL2 and CXCL12 genes with the susceptibility to breast cancer: a case-control study in China

**DOI:** 10.3389/fonc.2024.1475979

**Published:** 2024-12-05

**Authors:** Duanchong Zhao, Xingxing Yu, Hongli Huang, Shuqing Zou, Pingxiu Zhu, Yuxiang Lin, Mengjie Song, Fangmeng Fu, Haomin Yang

**Affiliations:** ^1^ Department of Epidemiology and Health Statistics, School of Public Health, Fujian Medical University, Fuzhou, China; ^2^ Department of Operation and Supervision, Jining Customs of the People’s Republic of China, Jining, China; ^3^ Department of Breast Surgery, Fujian Medical University Union Hospital, Fuzhou, China; ^4^ Breast Cancer Institute, Fujian Medical University, Fuzhou, China; ^5^ Department of Medical Epidemiology and Biostatistics, Karolinska Institutet, Stockholm, Sweden

**Keywords:** SNP, CCL2, CXCL12, breast cancer, chemokine

## Abstract

**Background:**

Chemokines are well-known for playing an essential role in the development of cancer. However, the association between SNPs in the CCL2 and CXCL12 genes and the susceptibility to breast cancer remains unclear.

**Methods:**

A case-control study was conducted in southeast China, including 1855 breast cancer patients and 1838 cancer-free controls. The association between single nucleotide polymorphisms (SNPs) in the CCL2 and CXCL12 genes and the susceptibility to breast cancer was investigated using logistic regression models. The association between plasma CCL2 and CXCL12 with breast cancer was further examined in 72 patients and 75 controls.

**Results:**

The CXCL12 SNP rs3740085 was associated with breast cancer in the additive model (OR=1.15, 95%CI=1.01-1.32), particularly in postmenopausal women. The association between rs1024611 in CCL2 and breast cancer was only found in women with a BMI of ≥24kg/m2. SNPs in the CCL2 gene were mainly associated with PR-positive breast cancer, whereas rs1144471 in CXCL12 was associated with ER-negative (OR=0.43, 95% CI=0.23-0.84), PR-negative (OR=0.38, 95% CI=0.19-0.74), and HER-2-positive (OR=1.27, 95% CI=1.03-1.56) breast cancer. The interaction between rs1801157 and rs3740085 in CXCL12 SNPs was statistically significant, and rs3740085 was also associated with breast cancer survival. Additionally, we found a strong association between plasma CXCL12 and breast cancer.

**Conclusion:**

CCL2 and CXCL12 SNPs are associated with breast cancer susceptibility in overweight and postmenopausal women, and the effect varies according to subtypes. The interaction of SNPs within CXCL12 gene and the association with breast cancer survival further suggest potential targets for improved risk assessment and treatment strategies.

## Introduction

Breast cancer is the second most common cancer, with an estimated 2.3 million new cases annually, constituting approximately 23.8% of female cancer cases worldwide (1). Despite Asian countries having had a lower incidence of breast cancer, the incidence rate has increased rapidly (2). Numerous efforts have been made to control breast cancer via advanced diagnostic techniques and novel anticancer drugs (3). However, the rates of incidence, mortality, and disability-adjusted life years (DALYs) attributed to breast cancer persistently escalate each year throughout the decades (4). In addition, recurrence and metastasis pose formidable challenges for breast cancer patients, emphasizing the urgent need for early prevention and treatment interventions to improve survival rates and overall quality of life.

Chemokines are currently promising candidates for early detection and biotherapeutic targets of breast cancer (5–7), which play an active role as immune mediators in tumor progression (8, 9). Genetic polymorphisms of CCL2 and CXCL12 may influence the binding of transcription factors to these genes, affecting promoter activity and gene transcription, and therefore they may have varying impacts on developing breast cancer.

CCL2 (CC chemokine ligand 2) has been shown to play a key role in the growth, invasion, and metastasis of breast cancer cells (5). Recently, a Mendelian Randomization analysis that systematically screened 41 cytokines identified that CCL2 was significantly associated with an increased risk of overall breast cancer, as well as ER-positive breast cancer (10). The CCL2-2518A/G (rs1024611) polymorphism increases risk of breast cancer in the Asian population (11, 12). However, the association of several other key polymorphisms, such as rs1024610, rs2530797, and rs3760396, with the susceptibility to breast cancer, and clinical subtypes of breast cancer remains inconclusive (13, 14).

Recent studies have indicated that the CXCL12 gene polymorphisms are associated with several cancer risks, including urogenital system cancers, lung cancer (15), nasopharyngeal carcinoma (16), and colorectal cancer (17). A recent study demonstrated that the polymorphism rs1801157 on the CXCL12 gene was associated with breast cancer and estrogen receptor positivity in Chinese (18). Additionally, the *in vitro* experiments confirmed that the GG genotype at rs1801157 could elevate the risk of breast cancer (19). However, more in-depth studies based on other tumor-related genetic polymorphisms of CXCL12, and their associations with breast cancer susceptibility are not yet fully understood.

In this study, we examined whether eight single nucleotide polymorphisms (SNPs) of CCL2 and CXCL12 influenced the susceptibility to breast cancer and analyzed their relationships with different subtypes of breast cancer. We further investigated their levels in plasma and breast cancer susceptibility.

## Materials and methods

### Study population

This hospital-based case-control study included 1855 breast cancer patients recruited from the Affiliated Union Hospital of Fujian Medical University between 2010 and 2016. For comparison, 1838 cancer-free subjects who underwent a health examination in the same hospital during the same period were frequency-matched with the patients by age groups (in 5-year groups). All participants were genetically unrelated Chinese Han residents of Fujian Province with no age limit. Each participant donated a 5-mL peripheral blood sample, and answered a questionnaire through face-to-face interviews. Demographic variables and risk factors including age, BMI, menstrual status, fertility status, number of abortions, and family history of breast or ovarian cancer were collected by trained interviewers. All cases and their immunohistochemical status of estrogen receptor (ER), progesterone receptor (PR), and human epidermal growth factor receptor-2 (HER2) were confirmed through pathological and histological examination. Lymph node metastases and tumor grade were also collected from the pathological reports. Follow-up of the patients was conducted routinely until 2017. This study and consent procedure were approved by the Ethical Committee of the Affiliated Union Hospital of Fujian Medical University, and all the procedures were performed according to the Declaration of Helsinki.

### Selection of SNPs

To investigate the potential association between SNPs in the CCL2 and CXCL12 genes, and susceptibility to breast cancer, we first focused on rs1024611 and rs1801157. These specific SNPs have shown significant association with breast cancer in other ethnic groups as reported in previous studies (11, 19, 20). We also included rs3760396 and rs3740085, which have been linked to cancer risk (14, 21). To increase the number of candidate SNPs, summary statistics of genome-wide association study for breast cancer in the Asian population were downloaded from the Breast Cancer Association Consortium (https://bcac.ccge.medschl.cam.ac.uk/). Those SNPs within the coding regions of CCL2 and CXCL12 genes, with an expansion to 500kb upstream and downstream, and meeting a significance threshold of P<0.05 were further selected. This resulted in the identification of 4 additional candidate SNPs: rs1024610, rs2530797, rs1144471, and rs2146807.

### DNA isolation and genotyping

Genomic DNA was extracted using a Whole-Blood DNA Extraction Kit (Bioteke, Beijing, China). A SNPscan Kit (Genesky Biotechnologies Inc., Shanghai, China) was used to analyze the genotypes. To conduct quality control, 10% of the samples were randomly selected and genotyped repeatedly in each run. The results indicated that 100% agreement was observed.

### Detection of plasma CCL2 and CXCL12

Plasma CCL2 and CXCL12 levels were measured in 72 cases and 75 controls using Luminex suspension chip detection technology. The Bio-Rad Cytokine Array kit was used following the manufacturer’s instructions for cytokine detection. After dilution, plasma samples were incubated on a microbead-embedded plate for 60 minutes at 23°C-25°C in the dark. Subsequently, the samples were treated with the biotinylated-detection antibody for 30 minutes. Streptavidin-PE was then added to each well to initiate color development, and the calibrated Luminex200 machine was used to quantify the values.

### Statistical analysis

Differences in the distributions of demographic characteristics, risk factors, and frequencies of alleles and genotypes between breast cancer cases and controls were evaluated by chi-square test. The relationship between the CXCL12 and CCL2 SNPs and susceptibility to breast cancer was assessed by calculating crude odds ratios (ORs) and 95% CIs using a logistic regression model. The multivariate model further adjusted for age, BMI, age at menarche, menopausal status, number of abortions, parity, history of hormone replacement therapy, age at birth of first child, family history of breast cancer/ovarian cancer, months of breastfeeding, and history of past breast surgeries. With this sample size, we had the power of 0.82 to detect an OR=1.2 under the additive genetic model, and 0.72 to detect OR=1.2 under the dominant genetic model (22). Gene-gene interactions were identified using the generalized multifactor dimensionality reduction (GMDR). Additionally, we conducted stratification analyses based on BMI, menopausal status, and age groups, and we also tested the associations between SNPs and different subtypes of breast cancer. The relationship between the CXCL12 and CCL2 SNPs and disease free survival of the breast cancer patients was further examined by Kaplan-Meier plot, together with a log-rank test. The association between plasma CCL2, CXCL12, and breast cancer susceptibility was assessed using logistic regression models. A P-value of <0.05 (two-sided) was considered as statistically significant. All statistical analyses were performed using R (version 4.1.3).

## Result

### Characteristics of the study population

The overall demographics of the participants are shown in **Supplementary Table S1**. There were no significant differences in the distribution of age, BMI, and menopausal status between cases and healthy controls. Compared with the control group, there were statistically significant differences in age of menopause, months of breastfeeding, history of hormone replacement therapy, age at first childbirth, parity, family history of breast or ovarian cancer, and the number of abortions. **Supplementary Table S2** has summarized some essential information for SNPs in CCL2 (rs1024610, rs1024611, rs2530797, and rs3760396) and CXCL12 (rs1144471, rs1801157, rs2146807, and rs3740085).

### Associations between CCL2 and CXCL12 SNPs and susceptibility to breast cancer

The associations between SNPs located in CCL2 and CXCL12 and susceptibility to breast cancer are presented in **Table 1**. No association was found among the three genetic models for SNPs in CCL2 gene. However, rs3740085 in CXCL12 showed a significant association with an increased risk of breast cancer (additive model: OR =1.15, 95% CI=1.01-1.32, dominant model: OR =1.18, 95% CI=1.01-1.37). The results were not statistically significant when we adjusted for other risk factors. GMDR analysis further suggested that the interaction of rs1801157 and rs3740085 in CXCL12 gene was significantly associated with susceptibility to breast cancer (**Supplementary Table S2**).

**Table 1:** Association of CCL2 and CXCL12 gene SNPs with breast cancer susceptibility.

SNP	Genetic Models	Genotypes	Controls	Cases	Crude OR (95% CI)	Adjusted OR^a^ (95% CI)
CCL2						
rs1024610	Additive	AA/TA/TT	1547/275/16	1558/283/14	1.00 (0.85-1.33)	1.00 (0.83-1.19)
	Dominant	AA/TA+TT	1547/291	1558/297	1.01 (0.85-1.21)	1.01 (0.83-1.22)
	Recessive	AA+TA/TT	1822/16	1841/14	0.87 (0.42-1.78)	0.88 (0.40-1.90)
rs1024611	Additive	GG/AG/AA	564/904/370	557/934/364	1.00 (0.91-1.10)	1.03 (0.93-1.13)
	Dominant	GG/AG+AA	564/1274	557/1298	1.03 (0.90-1.19)	1.07 (0.92-1.25)
	Recessive	GG+AG/AA	1468/370	1491/364	0.97 (0.82-1.14)	0.99 (0.83-1.18)
rs2530797	Additive	TT/CT/CC	1054/675/109	1068/659/128	1.02 (0.92-1.13)	1.05 (0.94-1.18)
	Dominant	TT/CT+CC	1054/784	1068/787	0.99 (0.87-1.13)	1.02 (0.88-1.18)
	Recessive	TT+CT/CC	1729/109	1727/128	1.18 (0.90-1.53)	1.25 (0.94-1.66)
rs3760396	Additive	GG/GC/CC	1491/324/23	1498/33819	1.01 (0.87-1.17)	1.01 (0.86-1.19)
	Dominant	GG/GC+CC	1491/347	1498/357	1.02 (0.87-1.21)	1.03 (0.86-1.23)
	Recessive	GG+GC/CC	1815/23	1855/19	0.82 (0.44-1.50)	0.78 (0.39-1.51)
CXCL12						
rs1144471	Additive	TT/TA/AA	1296/497/43	1332/483/39	0.94 (0.83-1.07)	0.93 (0.81-1.07)
	Dominant	TT/TA+AA	1296/540	1332/522	0.94 (0.82-1.08)	0.93 (0.79-1.08)
	Recessive	TT+TA/AA	1793/43	1815/39	0.90 (0.58-1.39)	0.89 (0.55-1.44)
rs1801157	Additive	CC/CT/TT	946/748/143	970/731/154	0.99 (0.90-1.10)	1.01 (0.91-1.13)
	Dominant	CC/CT+TT	946/891	970/885	0.97 (0.85-1.10)	0.99 (0.86-1.14)
	Recessive	CC+CT/TT	1694/143	1701/154	1.07 (0.85-1.36)	1.09 (0.84-1.41)
rs2146807	Additive	TT/CT/CC	1542/283/13	1571/276/8	0.93 (0.79-1.10)	0.95 (0.79-1.14)
	Dominant	TT/CT+CC	1542/296	1571284	0.94 (0.79-1.12)	0.97 (0.80-1.18)
	Recessive	TT+CT/CC	1825/13	1847/8	0.61 (0.24-1.44)	0.51 (0.18-1.34)
rs3740085	Additive	CC/CG/GG	1426/379/33	1382/432/39	**1.15 (1.01-1.32)**	1.08 (0.93-1.25)
	Dominant	CC/CG+GG	1426/412	1382/471	**1.18 (1.01-1.37)**	1.10 (0.93-1.30)
	Recessive	CC+CG/GG	1805/33	1814/39	1.18 (0.74-1.89)	1.00 (0.59-1.70)

^a^Adjusted for age, BMI, age at menarche, menopausal status, the number of miscarriages, parity, history of hormone replacement therapy, age at birth of first child, family history of Breast Cancer/ovarian cancer, months of breastfeeding, and history of past breast surgeries. Statistical significant results are in bold.

**Figure 1:**
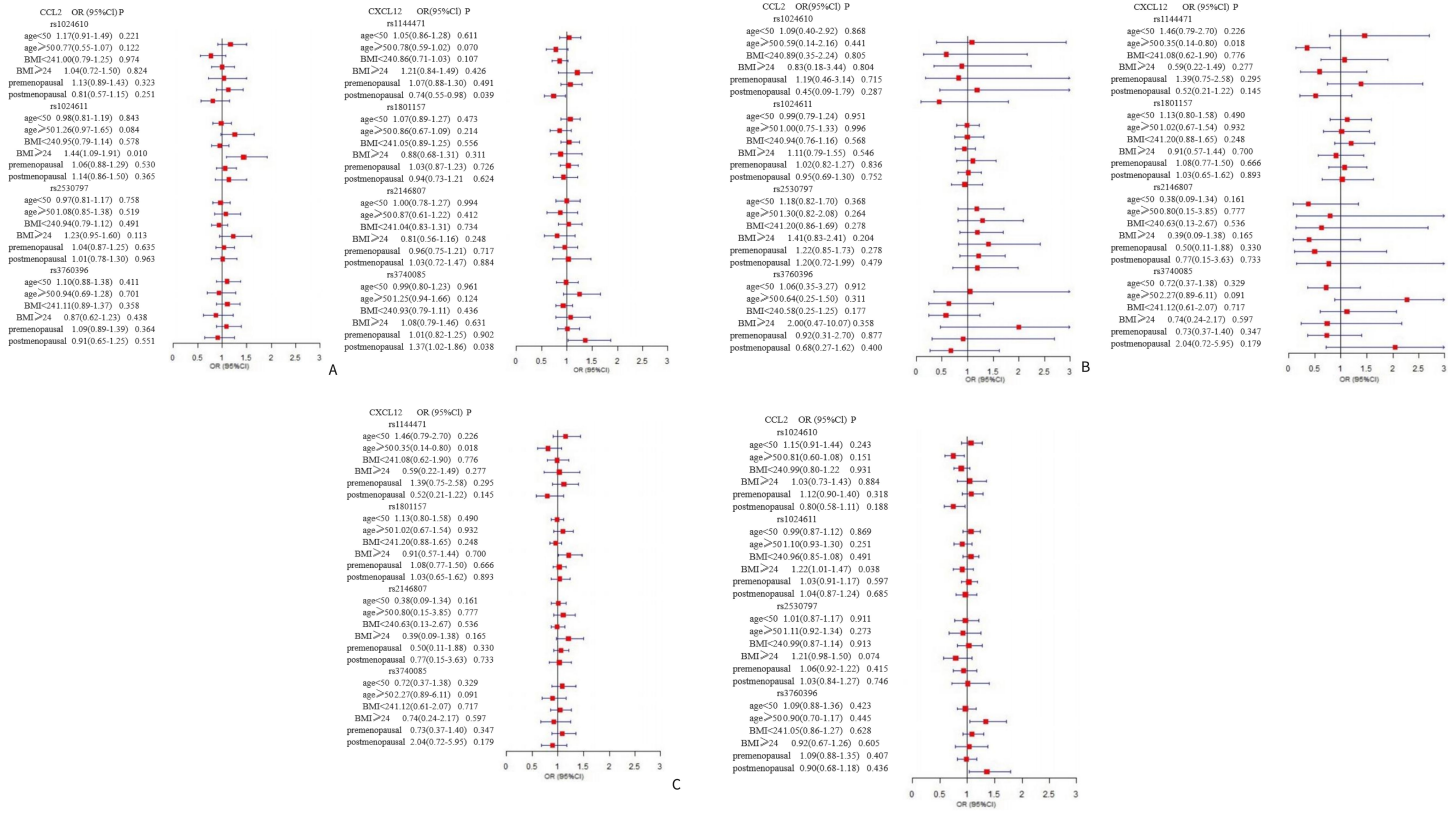
Forest plot for the association between CCL2 gene and CXCL12 gene polymorphisms and breast cancer susceptibility. The multivariate logistic regression was performed to identify eight SNPs on CCL2, and CXCL12 that may associate with breast cancer susceptibility. The analysis was stratified by age, BMI, and menopausal status adjusting for risk factors including age, BMI, age at menarche, menopausal status, the number of abortions, parity, history of hormone replacement therapy, age at birth of first child, family history of breast cancer/ovarian cancer, months of breastfeeding, and history of past breast surgeries. The panels **(A–C)** present the results of dominant, recessive, and additive model, respectively, for the association between CCL2 and CXCL12 gene polymorphisms and breast cancer susceptibility.

In the stratified analyses (**Figure 1**), rs1024611 in CCL2 was found to increase the susceptibility to breast cancer in the BMI≥24 (kg/m2) subgroup (dominant model: OR=1.44, 95% CI=1.09-1.91; addictive model: OR=1.22, 95% CI=1.01-1.47). For CXCL12, in the subgroup aged 50 years and older, an inverse association between rs1144471 and breast cancer was observed under the recessive model (OR=0.35, 95% CI=0.14-0.80) and additive model (OR=0.75, 95% CI=0.59-0.95), while rs3740085 showed a significant association with breast cancer under additive model (OR=1.34, 95% CI=1.05-1.71). Similar results were also observed among postmenopausal women.

### The associations between SNPs in CCL2, CXCL12, and breast cancer subtypes

For CCL2, there was no statistically significant difference in genotype distribution of the SNPs with ER status (**Table 2**). However, rs1024610, rs1024611 and rs2530797 were significantly associated with PR+ breast cancer (rs1024610: OR=1.30, 95% CI=1.01-1.69 for the additive model and OR=1.31, 95% CI=1.00-1.72 for the dominant model; rs1024611: OR=1.18, 95% CI=1.02-1.35 for the additive model, and rs2530797: OR= 1.17, 95% CI=1.00-1.37 for the additive model). The rs3760396 variant showed a significant association with HER-2+ breast cancer under the recessive model (OR=2.82, 95% CI= 1.12-7.21). The SNP rs1144471 located in CXCL12 showed a significant association with ER-, and PR- breast cancer, with OR of 0.43 (95% CI=0.23-0.84) and 0.38 (95% CI=0.19-0.74) for the recessive model, respectively. Additionally, rs1144471 was significantly associated with HER-2+ breast cancer under additive model (OR=1.27, 95% CI=1.03-1.56) and dominant model (OR=1.26, 95% CI=1.00-1.59).

**Table 2:** Association of CCL2 and CXCL12 gene SNPs with Breast Cancer subtypes.

SNP	Genetic Models	Genotype	ER			PR			HER-2		
			ER-(n=531)	ER+(n=1322)	OR^a^ (95% CI)	PR-(n=711)	PR+(n=1139)	OR^a^ (95% CI)	HER-2-(n=1234)	HER-2+(n=507)	OR^a^ (95% CI)
CCL2											
rs1024610	Additive	AA/TA/TT	456/71/3	1093/212/11	1.24 (0.95- 1.64)	611/96/3	936/186/11	**1.30 (1.01-1.69)**	1023/199/7	427/72/6	0.96 (0.73- 1.25)
	Dominant	AA/TA+TT	456/74	1093/223	1.26 (0.95- 1.70)	611/99	936/197	**1.31 (1.00-1.72)**	1023/206	427/78	0.91 (0.68- 1.21)
	Recessive	AA+TA/TT	527/3	1305/11	1.20 (0.40- 5.79)	707/3	1122/11	1.96 (0.60-8.78)	1222/7	499/6	2.18 (0.69- 6.67)
rs1024611	Additive	GG/AG/AA	163/277/90	390/653/273	1.11 (0.96- 1.29)	227/360/123	324/570/239	**1.18 (1.02-1.35)**	366/623/240	151/252/102	1.02 (0.88- 1.18)
	Dominant	GG/AG+AA	163/367	390/926	1.06 (0.85- 1.33)	227/483	324/809	1.20 (0.98- 1.48)	366/863	151/354	1.00 (0.80- 1.26)
	Recessive	GG+AG/AA	440/90	1043/273	1.29 (0.99- 1.69)	587/123	894/239	**1.28 (1.00-1.65)**	989/240	403/102	1.06 (0.81- 1.37)
rs2530797	Additive	TT/CT/CC	314/187/29	748/469/99	1.13 (0.96- 1.27)	425/244/41	635/411/87	**1.17 (1.00-1.37)**	700/440/89	302/174/29	0.89 (0.75- 1.06)
	Dominant	TT/CT+CC	314/216	748/568	1.11 (0.90- 1.37)	425/255	635/498	1.19 (0.97- 1.44)	700/529	302/203	0.89 (0.71- 1.11)
	Recessive	TT+CT/CC	501/29	1217/99	1.42 (0.93- 2.22)	669/41	1046/87	1.38 (0.93-2.06)	1140/89	476/29	0.78 (0.49- 1.19)
rs3760396	Additive	GG/GC/CC	425/97/8	1064/241/11	0.93 (0.74- 1.18)	580/120/10	907/217/9	1.06 (0.84- 1.33)	999/221/9	395/100/10	**1.26 (1.00- 1.60)**
	Dominant	GG/GC+CC	425/105	1064/255	0.95 (0.74- 1.24)	580/130	907/226	1.00 (0.86- 1.40)	999/230	395/110	1.22 (0.94- 1.58)
	Recessive	GG+GC/CC	522/8	1305/11	0.63 (0.25- 1.66)	700/10	1124/9	0.69 (0.26- 1.78)	1220/9	495/10	**2.82 (1.12- 7.21)**
CXCL12											
rs1144471	Additive	TT/TA/AA	382/130/18	944/350/21	0.90 (0.73- 1.10)	512/175/23	811/305/16	0.88 (0.73- 1.07)	903/306/20	348/141/15	**1.27 (1.03-1.56)**
	Dominant	TT/TA+AA	382/148	944/371	0.96 (0.76- 1.21)	512/198	811/321	0.95 (0.76- 1.18)	903/326	348/156	**1.26 (1.00-1.59)**
	Recessive	TT+TA/AA	512/18	1294/21	**0.43 (0.23- 0.84)**	687/23	1116/16	**0.38 (0.19-0.74)**	1209/20	489/15	1.90 (0.94-3.74)
rs1801157	Additive	CC/CT/TT	278/207/45	686/521/109	1.01 (0.86- 1.19)	385/264/61	578/463/92	1.08 (0.93- 1.26)	622/503/104	275/191/39	0.90 (0.76- 1.06)
	Dominant	CC/CT+TT	278/252	686/630	1.01 (0.83- 1.24)	385/325	578/555	1.14 (0.94- 1.39)	622/607	275/230	0.87 (0.70- 1.07)
	Recessive	CC+CT/TT	485/45	1207/109	1.02 (0.71- 1.48)	649/61	1041/92	1.00 (0.71- 1.42)	1125/104	466/39	0.90 (0.61- 1.32)
rs2146807	Additive	TT/CT/CC	450/79/1	1114/195/7	1.05 (0.80- 1.39)	602/106/2	961/166/6	1.03 (0.80- 1.33)	1045/176/8	424/81/0	1.03 (0.78- 1.36)
	Dominant	TT/CT+CC	450/80	1114/202	1.02 (0.77- 1.37)	602/108	961/172	1.00 (0.77- 1.32)	1045/184	424/81	1.09 (0.82- 1.45)
	Recessive	TT+CT/CC	529/1	1309/7	3.20 (0.56- 60.3)	708/2	1127/6	2.37 (0.53- 16.53)	1221/8	505/0	NA
rs3740085	Additive	CC/CG/GG	406/112/11	968/319/28	1.16 (0.94- 1.44)	540/151/18	833/278/21	1.11 (0.91- 1.35)	911/294/22	380/113/12	0.97 (0.78- 1.20)
	Dominant	CC/CG+GG	406/123	968/347	1.19 (0.94- 1.52)	540/169	833/299	1.18 (0.94- 1.47)	911/316	380/125	0.94 (0.73- 1.19)
	Recessive	CC+CG/GG	518/11	1287/28	1.07 (0.54- 2.29)	691/18	1111/21	0.75 (0.39- 1.47)	1205/22	494/12	1.31 (0.62-2.64)

^a^Adjusted for age, BMI, age at menarche, menopausal status, the number of miscarriages, parity, history of hormone replacement therapy, age at birth of first child, family history of Breast Cancer/ovarian cancer, months of breastfeeding, and history of past breast surgeries. Statistical significant results are in bold.

For lymph node metastasis, there was no clear association between CCL2, CXCL12 SNPs with lymph node status (**Table 3**). However, rs3760396 in CCL2 was inversely associated with grade III tumors (OR=0.72, 95%CI=0.52-0.99) in the dominant model.

**Table 3:** Association of CCL2 and CXCL12 gene SNPs with lymph node metastasis and histological grade of breast cancer.

SNP	Genetic Models	Genotype	Lymph node metastasis			Histological grade		
			LN metastasis- n=732	LN metastasis+ n=552	OR^a^ (95%CI)	I+II n=738	III n=458	OR^a^ (95%CI)
CCL2								
rs1024610	additive	AA/TA/TT	617/108/5	460/87/5	1.10 (0.82-1.47)	613/119/4	387/65/6	0.96 (0.70-1.30)
	dominant	AA/TA+TT	617/113	460/92	1.10 (0.80-1.50)	732/4	387/71	0.90 (0.65-1.26)
	recessive	AA+TA/TT	725/5	547/5	1.40 (0.39-5.09)	613/123	452/6	2.17 (0.61-8.57)
rs1024611	additive	GG/AG/AA	235/358/137	168/272/112	1.06 (0.90-1.25)	315/371/150	153/218/87	1.10 (0.82-1.47
	dominant	GG/AG+AA	235/495	168/284	1.05 (0.82-1.36)	686/150	153/305	0.81 (0.62-1.06)
	recessive	GG+AG/AA	593/137	440/112	1.12 (0.84-1.50)	315/521	371/87	0.89 (0.66-1.21)
rs2530797	additive	TT/CT/CC	418/266/46	316/194/42	1.03 (0.85-1.24)	416/265/55	269/158/31	1.03 (0.85-1.24)
	dominant	TT/CT+CC	418/312	316/236	0.94 (0.74-1.21)	416/320	269/189	0.90 (0.69-1.16)
	recessive	TT+CT/CC	684/46	510/42	1.26 (0.80-2.00)	681/55	427/31	0.83 (0.51-1.33)
rs3760396	additive	GG/GC/CC	586/139/5	452/95/5	0.89 (0.67-1.17)	577/154/5	385/68/5	0.85 (0.67-1.07)
	dominant	GG/GC+CC	586/144	452/100	0.86 (0.64-1.16)	577/159	385/73	**0.72 (0.52-0.99)**
	recessive	GG+GC/CC	725/5	547/5	1.34 (0.36-4.95)	734/5	453/5	1.61 (0.44-6.00)
CXCL12								
rs1144471	additive	TT/TA/AA	516/200/13	413/125/14	0.85 (0.67-1.07)	540/184/12	327/117/13	1.20 (0.94-1.53)
	dominant	TT/TA+AA	516/213	413/139	0.80 (0.61-1.04)	540/196	327/130	1.17 (0.89-1.54)
	recessive	TT+TA/AA	716/13	538/14	1.23 (0.55-2.75)	724/12	444/13	1.92 (0.83-4.59)
rs1801157	additive	CC/CT/TT	392/279/59	294/210/48	1.02 (0.85-1.22)	392/285/59	242/177/39	1.07 (0.88-1.30)
	dominant	CC/CT+TT	392/338	294/258	1.05 (0.83-1.82)	392/344	242/216	1.07 (0.84-1.38)
	recessive	CC+CT/TT	671/59	504/48	0.97 (0.63-1.48)	677/59	419/39	1.16 (0.74-1.81)
rs2146807	additive	TT/CT/CC	618/109/3	472/76/4	1.01 (0.74-1.37)	632/97/7	383/75/0	1.10 (0.80-1.50)
	dominant	TT/CT+CC	618/112	472/80	0.98 (0.71-1.36)	632/104	383/75	1.20 (0.86-1.68)
	recessive	TT+CT/CC	727/3	548/4	1.89 (0.41-9.70)	729/7	458/0	NA
rs3740085	additive	CC/CG/GG	554/163/12	407/135/10	1.06 (0.82-1.35)	551/173/12	343/106/8	1.00 (0.77-1.29)
	dominant	CC/CG+GG	554/175	407/145	1.08 (0.82-1.41)	551/185	343/114	0.99 (0.74-1.31)
	recessive	CC+CG/GG	717/12	542/10	0.90 (0.32-2.38)	724/12	449/8	1.13 (0.40-1.01)

^a^Adjusted for age, BMI, age at menarche, menopausal status, the number of miscarriages, parity, history of hormone replacement therapy, age at birth of first child, family history of Breast Cancer/ovarian cancer, months of breastfeeding, and history of past breast surgeries. Statistical significant results are in bold.

### The associations between SNPs in CCL2, CXCL12 and breast cancer survival

**Figure 2:**
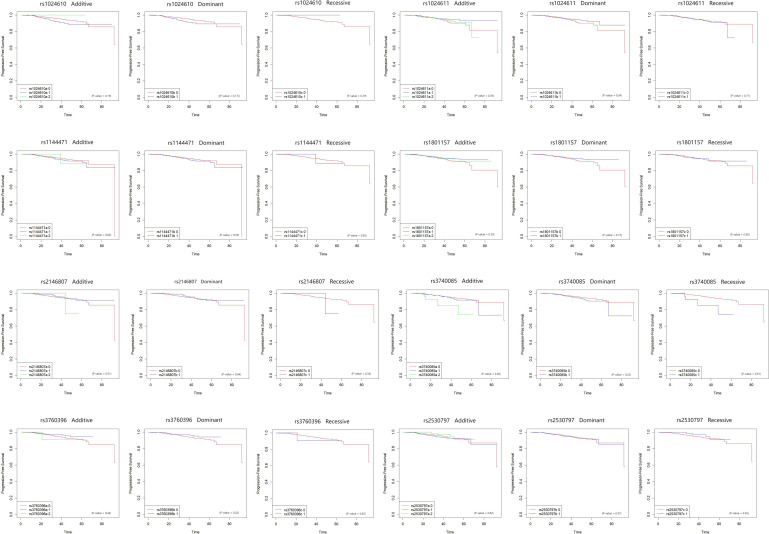
Kaplan-Meier plot for the association between CCL2 and CXCL12 SNPs and progression free survival in breast cancer patients. The associations between eight SNPs on CCL2 and CXCL12, and progression free survival of breast cancer patients were plotted by Kaplan-Meier method. Each of the SNP was analyzed by dominant, recessive, and additive models. Log-rank tests were used to assess the association. The X axis is months since diagnosis.

During the median follow-up time of 29 months, 69 patients died or suffered from distant metastasis. Among the 8 SNPs in the CCL2 and CXCL12 genes, rs3740085 in CXCL12 was associated with poor progression free survival of the breast cancer patients (p for log-rank test=0.02). Other SNPs did not show statistical significant results (**Figure 2**).

### Plasma levels of CCL2 and CXCL12 and the association with breast cancer

**Figure 3:**
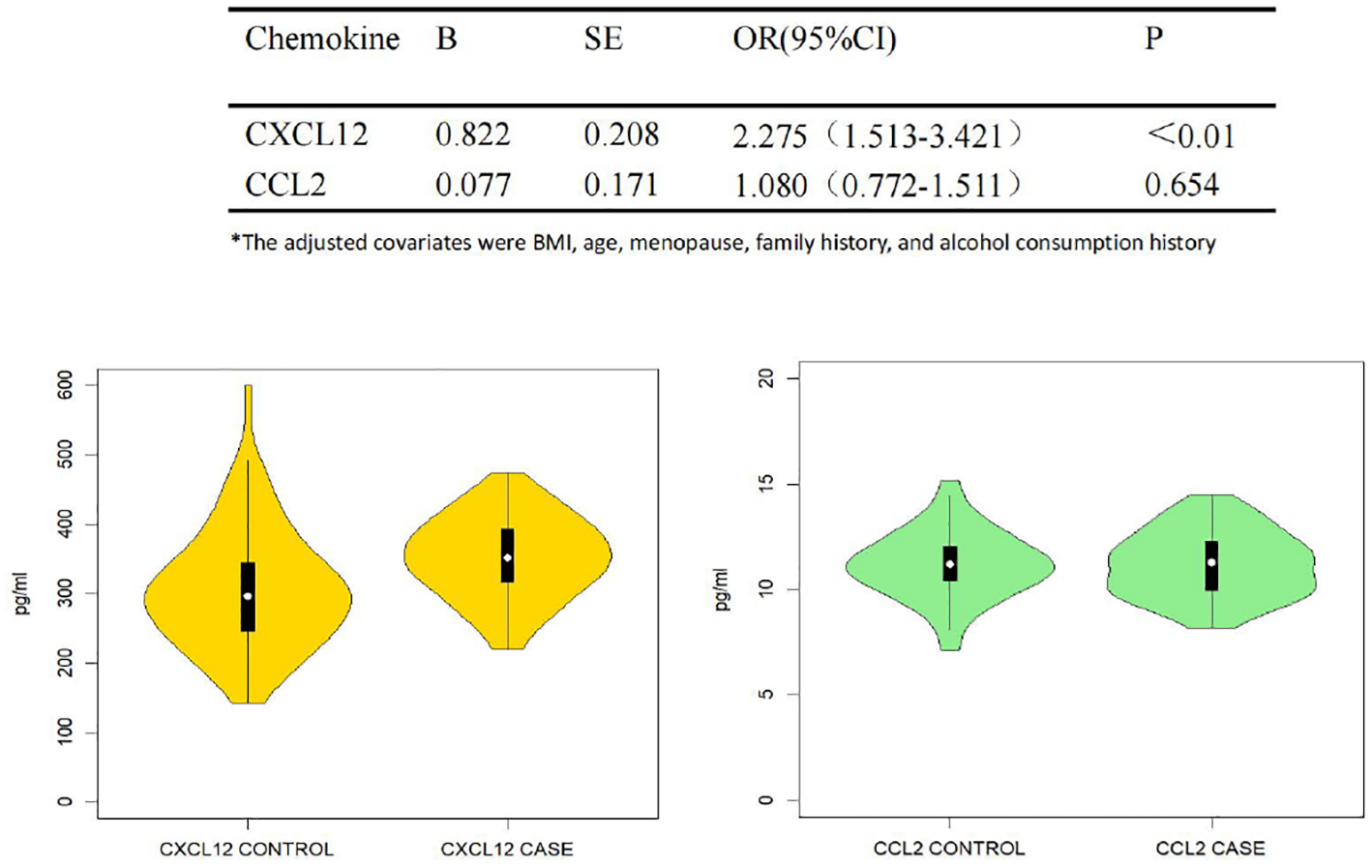
Plasma CCL2, CXCL12 and the association with breast cancer. Trilinear table with violin plots of plasma CCL2 and CXCL12 in correlation with breast cancer susceptibility. Multivariate logistic regression analysis was used, adjusted for BMI, age, menopausal status, and family history of breast cancer/ovarian cancer as covariates. Tables and images show the results of the association of CCL2 and CXCL12 with breast cancer susceptibility and the distribution of the two cytokines between the case and control groups, respectively.

**Figure 3** shows the plasma levels of CCL2 and CXCL12 in breast cancer patients compared to healthy individuals. There was a significant difference between cases and controls in plasma CXCL12 level, whereas we did not observe the association for CCL2.

## Discussion

In this study, we investigated the potential correlation between breast cancer susceptibility and eight specific SNPs within both the CCL2 and CXCL12 genes among the Chinese population. We found that the SNPs on the CCL2 gene showed no association with breast cancer risk in all genetic models. However, overweight women with AA and AG+AA genotypes in CCL2 SNP rs1024611 had a higher risk of breast cancer compared to those with GG. Also, Jia et al. (23) conducted meta-analyses on researches related to oral cancer, cervical cancer, breast cancer, and bladder carcinoma indicating that the GG genotype was associated with a reduced risk of overall cancers. We hypothesized that polymorphism of rs1024611 could modulate the expression of CCL2 and the recruitment of leukocytes to the inflammatory site, which are associated with the regulation of inflammation progression and the production of pro-inflammatory cytokines (24, 25). In addition to its role of immunosuppression via regulating PD-1 expression in macrophages surrounding breast cancer cells (26), CCL2 may also influence breast cancer cell proliferation and cell-cycle progression through SRC and PKC activation (27). The significant correlation between the polymorphism in the CCL2 gene and breast cancer observed in individuals with a BMI≥24 kg/m2 may result from a potential mechanism involving adipocytes (28) within breast tissues, especially prevalent in obese populations. These adipocytes may initiate the recruitment and activation of macrophages through a CCL2 signaling pathway. This activation could, in turn, promote stromal vascularization and angiogenesis, preparing the microenvironment for neoplastic transformation and accelerating breast oncogenesis even before cancer formation takes place.

In the current study, SNPs rs1024610, rs1024611, and rs2530797 in CCL2 were significantly associated with the PR status, while rs3760396 was correlated with the Her2 status and tumor grade. It was reported that estrogen upregulated TWIST via PI3K/AKT/NF-kappa B signaling to modulate the physiological role of the CCL2-CCR2 biological axis, which could be a novel therapeutic target for hormone-dependent breast cancer (29). Tewari et al. (30) also stated that CCL2 showed a significant association with PR, which is consistent with our results. Furthermore, it was demonstrated that both the transcription and secretion levels of CCL2 were elevated in the triple-negative breast cancer cell line (31). However, the conclusion that the expression levels of CCL2 are influenced by hormones and epidermal growth factor receptors, affecting the development and progression of breast cancer, should be interpreted with caution due to factors such as sample size, different genetic backgrounds, ethnicity, and experimental methods.

Our results showing that women with breast cancer had higher plasma levels of CXCL12, compared to healthy individuals, was confirmed in another population-based study. With regard to the relationships between CXCL12 SNPs and breast cancer, we additionally discovered that rs1144471 and rs3740085 were associated with susceptibility to breast cancer in the Chinese population, particularly in postmenopausal individuals. Further analyses of CXCL12 associated with breast cancer subtypes revealed that the homozygous mutation AA genotype in SNP rs1144471 was associated with ER-negative and PR-negative breast cancer but not with HER-2-positive breast cancer. In addition, rs3740085 was also associated with breast cancer survival. According to the latest study, CXCL12 may primarily promote the epithelial-mesenchymal transition of Her2-positive cancer cells through interaction with the receptor CXCR4 (32). Wei Wu et al. (33) further found that the expression of CXCL12 mRNA and CXCL12 protein is significantly higher in Her2 overexpressing breast cancer compared to Luminal A and Luminal B subtypes. Blocking the CXCL12/CXCR4 pathway may suppress the growth of breast cancer cells by reducing the expression of proteins that facilitate the G2-M phase transition (PLK1, Myt1, and cyclin B1), and then disrupting mitotic processes (34). Furthermore, other studies have suggested that high levels of CXCL12 expression are often found in patients with breast cancer lymph node and brain metastases, which correlates with poorer overall survival (35–37). Altogether, CXCL12 may provide a potential therapeutic target for breast cancer, which could pave the way for innovative approaches to breast cancer therapies.

Numerous previous studies have focused on the association between the rs1801157 polymorphism and breast cancer, but the findings have not yet reached a conclusive agreement. Several studies have reported the polymorphism of rs1801157 associated with breast cancer (38, 39). In addition, meta-analyses indicated that this polymorphism increased the risk of breast cancer in allelic, homologous, heterologous, recessive, and dominant genetic models (40). However, other studies do not support a correlation between them (41–43), which is consistent with the results of our study. In addition, the influence of the rs1801157 polymorphism on expression levels of CXCL12 remains controversial. A study conducted on the Brazilian population found that the rs1801157 allele A was associated with low expression of CXCL12 in peripheral blood in ER-positive breast cancer (43). The role of rs1801157 may therefore need further studies.

Furthermore, the current study showed that the interaction between rs1801157 and rs3740085 was significantly associated with breast cancer. However, there was no significant correlation of CXCL12 SNPs rs1801157 with breast cancer. A study on the combined effects of SNPs in CXCL12 biological axis-related genes revealed that women with the CC/GG genotype in rs2228014-rs1801157 (CXCR4-CXCL12) exhibited a significantly lower risk of breast cancer, whereas there was no relationship between individual SNP and breast cancer (44). It is supposed that certain SNPs, which either have no direct effect on cancer risk or whose direct effects are too small to be detected, may interact with other SNPs to contribute to changes in cancer risk.

### Strengths and limitations

This case-control study emphasized the importance of having sufficient sample sizes to achieve a thorough assessment of the relationship between the CCL2, and CXCL12 SNPs and breast cancer. This is crucial because most of the genetic variants typically have a low or moderate impact on cancer susceptibility (45). We therefore added the analysis of plasma CCL2 and CXCL12 which confirmed the association with breast cancer. Additionally, the cases included in our study were all newly diagnosed and had undergone a clear pathological diagnosis, which is advantageous for controlling prevalence-incidence bias. Some limitations in our study must be acknowledged. Firstly, bias may have occurred in our analysis due to the hospital-based study. Secondly, in the stratified analysis of this study, the sample size of some research subgroups was moderate, which may affect the statistical power. In the future, these results should be confirmed with a higher quality population.

## Conclusion

In conclusion, the present study highlights that the CCL2 and CXCL12 SNPs are linked to breast cancer susceptibility, especially in overweight and postmenopausal Chinese women. Moreover, CCL2 SNPs were more likely to be associated with PR-positive breast cancer, while CXCL12 SNPs were associated with three main subtypes of breast cancer. The interaction between the CXCL12 SNPs and the association with breast cancer survival further suggest potential targets for better detection and treatment opportunities for breast cancer.

## Data Availability

The raw data supporting the conclusions of this article will be made available by the authors, without undue reservation.
