# Supplementary material for: Association of the SNPs in CCL2 and CXCL12 genes with the susceptibility to breast cancer: a case-control study in China
Source: Front Oncol. 2024 Dec 5;14:1475979. doi: 10.3389/fonc.2024.1475979 (PMC11655334; doi:10.3389/fonc.2024.1475979)
Supplement: Supplementary file 1 [file Table1.docx]

Supplementary Table 1 Distribution of selected demographic variables and risk factors in breast cancer cases and controls

| Variable | Cases(n=1838) | Controls(n=1855) | *χ*2 | Pa |
| --- | --- | --- | --- | --- |
| Age, mean (±SD) | 47.06±10.73 | 47.12±10.68 |  | 0.707b |
| BMI (kg/m2) |  |  | 3.805 | 0.149 |
| <18.5 | 104 | 116 |  |  |
| 18.5-24.9 | 1323 | 1371 |  |  |
| ≥25 | 411 | 368 |  |  |
| Menopausal status |  |  | 1.562 | 0.211 |
| Pre-menopausal | 1150 | 1201 |  |  |
| Post-menopausal | 603 | 576 |  |  |
| Menarche age(years) |  |  | 47.684 | <0.001 |
| <12 | 14 | 25 |  |  |
| 12- 15 | 953 | 1156 |  |  |
| >15 | 871 | 674 |  |  |
| Months of breastfeeding |  |  | 3.929 | 0.047 |
| <4 | 260 | 306 |  |  |
| ≥4 | 1578 | 1549 |  |  |
| History of hormone replacement therapy |  |  | 22.409 | <0.001 |
| No | 1797 | 1759 |  |  |
| Yes | 41 | 96 |  |  |
| Parity |  |  | 50.357 | <0.001 |
| 0 | 79 | 88 |  |  |
| 1-2 | 1343 | 1514 |  |  |
| Family history of breast / ovarian cancer |  |  | 86.709 | <0.001 |
| No | 1809 | 1703 |  |  |
| Yes | 29 | 152 |  |  |
| Age at first childbirth (years) |  |  | 41.938 | <0.001 |
| <25 | 977 | 789 |  |  |
| 25-29 | 628 | 779 |  |  |
| ≥30 | 154 | 199 |  |  |
| History of breast surgeries |  |  | 42.175 | <0.001 |
| No | 1768 | 1687 |  |  |
| Yes | 70 | 168 |  |  |
| The number of abortions |  |  | 412.488 | <0.001 |
| <2 | 1769 | 1326 |  |  |
| ≥2 | 69 | 524 |  |  |

a: chi-square test. b: Two-sided T-test
