# Supplementary material for: Association of the SNPs in CCL2 and CXCL12 genes with the susceptibility to breast cancer: a case-control study in China
Source: Front Oncol. 2024 Dec 5;14:1475979. doi: 10.3389/fonc.2024.1475979 (PMC11655334; doi:10.3389/fonc.2024.1475979)
Supplement: Supplementary file 2 [file Table2.docx]

Supplementary Table 2 Primary information for SNPs in CCL2 and CXCL12

| Gene | Genotyped SNPs | Chromosome Position | Allele Major/Minor | MAF^a^ | Pb-HWE |
| --- | --- | --- | --- | --- | --- |

|  |  |  |  | Controls Cases |  |
| --- | --- | --- | --- | --- | --- |
| CCL2 | rs1024610 rs1024611 rs2530797  rs3760396 | chr17:32580231 chr17:32579788 chr17:32586094  chr17:32581441 | A/T  G/A  T/C  G/C | \| 0.084 \| 0.084 \| \| --- \| --- \| \| 0.447 \| 0.448 \| \| 0.243 \| 0.247 \| \| 0.101 \| 0.101 \| | 0.358  0.814  0.949  0.247 |
|  |  |  |  |  |  |
| CXCL12 | rs1144471 rs1801157 rs2146807  rs3740085 | chr10:44869625 chr10:44868257 chr10:44813738  chr10:44867769 | T/A  C/T  T/C  C/G | \| 0.159 \| 0.151 \| \| --- \| --- \| \| 0.281 \| 0.280 \| \| 0.084 \| 0.079 \| \| 0.121 \| 0.138 \| | 0.601  0.818  1.000  0.188 |

a: MAF, minor allele frequency. b: HWE, Hardy–Weinberg equilibrium.
