# Supplementary material for: Association of the SNPs in CCL2 and CXCL12 genes with the susceptibility to breast cancer: a case-control study in China
Source: Front Oncol. 2024 Dec 5;14:1475979. doi: 10.3389/fonc.2024.1475979 (PMC11655334; doi:10.3389/fonc.2024.1475979)
Supplement: Supplementary file 3 [file Table3.docx]

Supplementary Table 3 The gene–gene interaction analysis for CCL2 and CXCL12

| model | Average precision on the training set | Test set average accuracy | sign test(P) | Ten-fold cross-validation agreement rate |
| --- | --- | --- | --- | --- |
| Between CCL2 gene SNPs |  |  |  |  |
| rs1024611 | 0.5103 | 0.4863 | 1(0.999) | 4/10 |
| rs1024611 rs2530797 | 0.5158 | 0.4884 | 1(0.999) | 7/10 |
| rs1024610 rs1024611 rs2530797 | 0.5176 | 0.4796 | 1(0.999) | 4/10 |
| rs1024610 rs1024611 rs2530797 rs3760396 | 0.5192 | 0.4765 | 1(0.999) | 10/10 |
| CXCL12 gene between SNPs |  |  |  |  |
| rs3740085 | 0.5158 | 0.5159 | 8(0.055) | 10/10 |
| rs1801157 rs3740085 | **0.5196** | **0.5150** | **9(0.011)** | **9/10** |
| rs1144471 rs2146807 rs3740085 | 0.5240 | 0.5111 | 7(0.172) | 9/10 |
| rs1144471 rs1801157 rs2146807 rs3740085 | 0.5288 | 0.4914 | 3(0.945) | 10/10 |
| Between CCL2 and CXCL12 genes |  |  |  |  |
| rs3740085 | 0.5158 | 0.5159 | 8(0.055) | 10/10 |
| rs2530797 rs3740085 | 0.5211 | 0.5010 | 7(0.172) | 4/10 |
| rs1024611 rs1801157 rs3740085 | 0.5292 | 0.4866 | 2(0.989) | 3/10 |
| rs3760396 rs11444471 rs1801157 rs3740085 | 0.5399 | 0.5050 | 5(0.623) | 8/10 |
